# Recent developments and future directions of first-line systemic therapy combined with immunotherapy for advanced or metastatic urothelial carcinoma: a historical perspective on treatment evolution

**DOI:** 10.1007/s10147-024-02526-y

**Published:** 2024-06-08

**Authors:** Atsunari Kawashima, Yu Ishizuya, Yoshiyuki Yamamoto, Taigo Kato, Koji Hatano, Norio Nonomura

**Affiliations:** Department of Urology, Graduate School of Medicine, Osaka University, 2-2 Yamadaoka, Suita City, Osaka 565-0871 Japan

**Keywords:** Urothelial carcinoma, ADC, Immunotherapy, Chemotherapy, First line

## Abstract

Urothelial carcinoma presents significant treatment challenges, especially in advanced stages. Traditionally managed with platinum-based chemotherapy, the advent of immunotherapies, particularly immune checkpoint inhibitors, has revolutionized urothelial carcinoma treatment. This review explores the evolution of urothelial carcinoma management, focusing on the transition from immune checkpoint inhibitors monotherapy to innovative combination therapies. Pembrolizumab, following the KEYNOTE-045 trial, emerged as a pivotal ICI in pretreated metastatic urothelial carcinoma, outperforming traditional chemotherapy. However, limitations surfaced in untreated metastatic urothelial carcinoma patients, particularly in those with low PD-L1 expression, as evidenced by trials like IMvigor130 and KEYNOTE-361. These challenges led to the exploration of combination therapies, including immune checkpoint inhibitors with platinum-based chemotherapy, tyrosine kinase inhibitors, and antibody–drug conjugates. Notably, the CheckMate 901 trial demonstrated improved outcomes with a nivolumab–chemotherapy combination. A significant breakthrough was achieved with the combination of enfortumab vedotin, an antibody–drug conjugates, and pembrolizumab, setting a new standard in first-line treatment for locally advanced or metastatic urothelial carcinoma. Future directions involve further exploration of antibody–drug conjugates and immune checkpoint inhibitors, as seen in the TROPHY-U-01 and TROPiCS-4 trials. The review concludes that the locally advanced or metastatic urothelial carcinoma treatment landscape is rapidly evolving, with combination therapies offering promising avenues for improved patient outcomes, signaling a new era in urothelial carcinoma management.

## Introduction

Urothelial carcinoma (UC), which originates from the urothelium, can develop anywhere from the renal pelvis, ureter, or bladder to the urethra. Globally, bladder cancer ranks as the tenth most diagnosed cancer, with UC being its predominant subtype [1]. Over the years, the incidence of UC has been on a steady rise, making it a significant public health concern [2].

For decades, the management of UC, especially in its advanced and metastatic stages, has been dominated by platinum-based chemotherapy [3, 4]. Although these regimens have provided some benefit, the outcomes for many patients, particularly those who are chemotherapy resistant or ineligible, remain suboptimal [5, 6]. The 5-year survival rate for metastatic UC (mUC) hovers around a mere 5–6%, highlighting the dire need for more effective therapeutic strategies [2].

In recent years, a surge of interest in understanding the interactions between tumors and the immune system [7, 8] has culminated in the development of immunotherapies aiming to harness the body’s immune system to recognize and combat cancer cells. Immune checkpoint inhibitors (ICIs), a class of immunotherapies, have emerged as a beacon of hope in the oncology community. Their potential to revolutionize the treatment landscape for a variety of cancers, including UC, has been the subject of considerable research and clinical trials.

The promise of ICIs in UC treatment offers not only improved survival outcomes but also a better quality of life [9, 10]. As we delve deeper into this review, the current status, challenges, and future prospects of immunotherapy for UC will be explored to provide insights into this rapidly evolving field.

## Establishment of ICI monotherapy for pretreated mUC patients

The journey of immunotherapy in the second-line treatment of locally advanced or metastatic urothelial carcinoma (la/mUC) reflects a significant paradigm shift in cancer care, marking the transition from traditional chemotherapy to targeted immune therapies [11]. This evolution began with the initial introduction of immunotherapies in this setting and has since progressed to a more established and nuanced understanding of their application (Table 1).

**Table 1:** Results of clinical trials of ICI monotherapy as first or second line treatment for locally advanced or metastatic urothelial carcinoma patients

Target	Regimen	Trial	Phase	Number of patients	Median PFS (months)	Median OS (months)	ORR (%)	Treatment related severe AE (≥ Grade 3) (%)
Second line								
Anti PD-L1	Atezolizumab	IMvigor211	III	467	N/A	8.6	13.0	20.0
	Avelumab	JAVELIN solid tumor	Ib	249	1.6	7.0	16.5	8.0
	Durvalumab	NCT01693562	I/II	191	1.5	18.2	17.8	6.8
Anti PD-1	Pembrolizumab	KEYNOTE-045	III	270	2.1	10.3	21.1	15.0
	Nivolumab	CheckMate 275	II	270	1.9	8.6	20.7	24.8
		CheckMate 032	II	78	2.8	9.9	25.6	26.9
First line								
Anti PD-L1	Atezolizumab	IMvigor210	II	119	2.7	15.9	23.0	16.0
		IMvigor130	III	362	N/A	15.7	23.0	15.0
	Durvalumab	DANUBE	III	346	2.3	13.2	26.0	21.0
Anti PD-1	Pembrolizumab	KEYNOTE-052	II	370	2.5	11.3	28.6	19.0
		KEYNOTE-361	III	307	3.9	15.6	30.3	27.1
		LEAP-011	III	223	4.0	13.8	26.5	14.0

*ICI* immune checkpoint inhibitor, *PFS* progression free survival, *OS* overall survival, *ORR* overall response rate, *AE* adverse event, *PD-1* programmed death 1, *PD-L1* programmed death ligand 1

The role of pembrolizumab in this transition became particularly prominent following the results of the KEYNOTE-045 trial [12]. This phase III study of anti PD-1 therapeutic antibody was a milestone, demonstrating the efficacy of pembrolizumab over traditional chemotherapy options of paclitaxel, docetaxel, or vinflunine in patients with la/mUC who had progressed after platinum-based chemotherapy. The trial reported a median overall survival (mOS) of 10.3 months for pembrolizumab as compared to 7.4 months for chemotherapy, establishing it as a new standard of care. Long-term follow-up results further solidified its position by confirming superior survival rates and median duration of response. It also showed a certain benefit in patients with poor performance status and the elderly, to whom chemotherapy was considered difficult to administer in the past, and it contributed to broadening the treatment options [13].

In contrast, several other drugs targeting the same PD-1/PD-L1 receptors did not show positive test results. Although anti-PD-L1 therapeutic antibody atezolizumab, which was initially approved by the U.S. Food & Drug Administration (FDA) in 2016 based on the IMvigor210 trial [14], marked another early success story in the adoption of immunotherapies, the subsequent phase III IMvigor211 trial did not show significant improvement in overall survival (OS) or response rate in a specific patient subgroup [15]. Three other anti PD-L1 therapeutic antibodies, avelumab [16, 17], durvalumab [18], and anti-PD-1 therapeutic antibody nivolumab [19–21], also showed no significant improvement in phase III trials.

The introduction and establishment of immunotherapies as second-line treatments for la/mUC represent a significant advancement in general practice [22, 23]. This underscores a shift from conventional chemotherapies to more targeted approaches that harness the body’s immune system, albeit with ongoing challenges and education in optimizing their efficacy and understanding their long-term impacts.

## Limitations of ICI monotherapy for untreated mUC patients

The application of immunotherapy as a primary treatment for la/mUC in cisplatin-ineligible patients has faced significant challenges, as evidenced by results from several key clinical trials (Table 1). Initially, the 2017 FDA approval of atezolizumab and pembrolizumab based on the phase II IMvigor210 [24] and KEYNOTE-052 [25] trials marked a hopeful advancement. In IMvigor210, atezolizumab showed an objective response rate (ORR) of 23%, mOS of 15.9 months, and median progression-free survival (mPFS) of 2.7 months. Meanwhile, KEYNOTE-052 reported an ORR at 28.6% and mOS at 11.3 months for pembrolizumab. Both trials noted higher efficacy in patients with higher PD-L1 expression.

In this setting, optimism was tempered by safety concerns. In KEYNOTE-052, 67% of patients experienced adverse events (AEs) of any grade, with about 21% suffering from ≥ grade 3 AEs, including colitis and pneumonitis [26]. These findings highlighted the need for cautious patient selection and management.

The situation further evolved in May 2018 when the European Medicines Agency and the FDA issued safety alerts based on early data from the phase III IMvigor130 [27] and KEYNOTE-361 [28] trials. These trials indicated a decreased OS for patients with tumors showing low PD-L1 expression who received ICIs as monotherapy as compared to those on platinum-based therapy, although both agents are only recommended for la/mUC patients who are ineligible for any platinum-based chemotherapy [29, 30].

Although initial results from ICIs in la/mUC were promising, subsequent trials have revealed limitations, particularly in patients with low PD-L1 expression.

## Development of combination therapies with ICIs for untreated mUC patients

### Maintenance therapy with ICIs after platinum-based induction chemotherapy

Induction chemotherapy for la/mUC is effective in some patients but does not last long [3, 4]. Thus, maintenance therapy with various agents including chemotherapy [31–36], tyrosine kinase inhibitors [37, 38] and poly ADP ribose polymerase inhibitors [39, 40] has been investigated and reported to maintain the therapeutic effect. Although a certain efficacy is reported in retrospective analysis, the actual evidence for efficacy remains unclear. The phase III JAVELIN Bladder 100 trial, which established avelumab as a maintenance therapy for la/mUC after platinum-based chemotherapy, represents a significant positive advancement in the treatment of this condition (Table 2) [41]. The trial results showing a mOS of 23.8 months for avelumab versus 15.0 months for the control group are indeed noteworthy and highlight the potential of immunotherapy in extending survival in la/mUC patients [42].

**Table 2:** Combination therapies with ICIs as first line treatment for locally advanced or metastatic urothelial carcinoma patients

Regimen	Control	Trial	Phase	ORR (%)	Median OS (months)	HR	95% CI		*p* value	Treatment related severe AE (≥ Grade 3) (%)
							Lower	Higher		
Maintenance Therapy after induction chemotherapy										
Avelumab	BSC	JAVELIN Bladder 100	III	9.7	23.8	0.76	0.63	0.91	0.0036	19.5
Pembrolizumab	BSC	NCT02500121	II	23.0	22.0	0.91	0.52	1.59	0.7477	59.0
ICIs / ICIs										
1. Durvalumab	GEM/Platinum (CDDP 56%)	DANUBE	III	36.0	15.1	0.85	0.72	1.02	0.075	29.0
2. Tremelimumab										
TKIs / ICIs										
1. Pembrolizumab	Pembrolizumab	LEAP-011	III	33.1	11.2	1.14	0.87	1.48	N/A	51.0
2. Lenvatinib										
Chemotherapy / ICIs										
1. Atezolizumab	GEM/Platinum (CDDP 34%)	IMvigor130	III	47.0	16.0	0.83	0.69	1.00	0.027	81.0
2. GEM/Platinum (CDDP 30%)										
1. Pembrolizumab	GEM/Platinum (CDDP 44%)	KEYNOTE-361	III	55.0	17.0	0.86	0.72	1.02	0.0407	73.0 ≤
2. GEM/Platinum (CDDP 44%)										
1. Nivolumab	GEM/CDDP	CheckMate 901	III	57.6	21.7	0.78	0.63	0.96	0.02	61.8
2. GEM/CDDP										
ADCs / ICIs										
1. Pembrolizumab	GEM/Platinum (CDDP 54.3%)	KEYNOTE-A39 EV-302	III	67.7	31.5	0.47	0.38	0.58	< 0.00001	55.9
2. Enfortumab vedotin										

*ICIs* immune checkpoint inhibitors, *ORR* overall response rate, *OS* overall survival, *HR* hazard ratio, *CI* confidence interval, *AE* adverse event, *BSC* best supportive care, *GEM* gemcitabine, *CDDP* cisplatin, *TKIs* tyrosine kinase inhibitors, *ADCs* antibody–drug conjugates

However, alongside these positive outcomes are methodological aspects of the trial that warrant consideration, such as the allowance of a range of 4–6 courses of induction chemotherapy prior to avelumab treatment. For instance, in a phase II trial using pembrolizumab in a comparable setting, significant differences in progression-free survival (PFS) were observed, but not in OS, after the implementation of a crossover from the control to the treatment arm [43]. This result suggested that there might be no difference between early maintenance therapy and delayed treatment. As a result, in real-world practice, the difference between switching to avelumab with SD or higher after 4 courses and using pembrolizumab after 6 courses with PD could not be clarified, resulting in the emergence of a trend toward earlier use of avelumab.

Another problem is that switch maintenance immunotherapy may lead to rapid deterioration of disease [44], so-called hyper-progressive disease (HPD) [45, 46]. Especially in UC, it has been reported that among several carcinomas, HPD is more likely to occur with immunotherapy than with chemotherapy [47]. Risk factors for HPD are reported to include higher age (> 65 years) [48], female [49], liver metastases and high serum LDH [50], MDM2/MDM4 amplification, and EGFR aberrations [51]. Therefore, clinicians need to be aware of the existence of disadvantaged patient groups in parallel with the identification of patients who will benefit from avelumab therapy [52].

This aspect highlights the need to carefully interpret survival benefits and consider the potential impact on patient outcomes in clinical practice by balancing the positive results of a trial with an understanding of its design limitations.

### Combination of several ICIs for untreated mUC patients

The phase III DANUBE trial, exploring the ICI/ICI combination of durvalumab and tremelimumab (anti CTLA-4 therapeutic antibody), did not meet its primary endpoints (Table 2) [53]. The study failed to show significant improvement in mOS with durvalumab monotherapy or combination therapy versus chemotherapy. An exploratory analysis in cisplatin-ineligible patients suggested a slight yet nonstatistically significant improvement in mOS for the ICI/ICI combination.

### Combination of tyrosine kinase inhibitors and ICIs for untreated UC patients

Recently, the LEAP-011 study, a phase 3 clinical trial, was reported for patients with la/mUC faced with limited treatment options, particularly those ineligible for any platinum-based chemotherapy (Table 2) [54]. This trial was groundbreaking in its approach as the first to combine lenvatinib, a multikinase inhibitor targeting VEGF receptors, fibroblast growth factor receptor (FGFR) receptors, and other receptors and oncogenes, with an immunotherapy agent, pembrolizumab. The rationale behind this combination was based on the understanding that VEGF upregulation plays a crucial role in angiogenesis in solid tumors, and alterations in the *FGFR* gene are common in UC patients.

In the trial, 487 patients were randomly assigned to receive either the combination of lenvatinib plus pembrolizumab or placebo plus pembrolizumab. However, the results did not show an advantage of the combination therapy over pembrolizumab alone. The mPFS was 4.5 months in the combination arm versus 4.0 months in the pembrolizumab arm, and the mOS was 11.8 months versus 12.9 months, respectively. Additionally, the incidence of grade 3–5 AEs was higher in the combination therapy group.

Findings from the LEAP-011 study indicated that the combination of lenvatinib and pembrolizumab did not improve outcomes compared to pembrolizumab alone in advanced UC, and thus, the trial was terminated early on this basis. This outcome underscores the ongoing challenge in developing effective and tolerable treatment regimens for this patient population and highlights the need for continued research into novel therapeutic combinations.

### Combination of chemotherapy and ICIs for untreated UC patients

The IMvigor130 and KEYNOTE-361 phase III trials, which evaluated the combination of immunotherapy with chemotherapy in the treatment of la/mUC, demonstrated limited efficacy in improving patient outcomes compared to chemotherapy alone (Table 2).

In the IMvigor130 trial [27], 1213 la/mUC patients were randomized to receive either atezolizumab with platinum-based chemotherapy (gemcitabine plus cisplatin or carboplatin), atezolizumab alone, or platinum-based chemotherapy alone. Although the addition of atezolizumab to chemotherapy showed a marginal improvement in mPFS (8.2 vs. 6.3 months, *p* = 0.014), the critical co-primary endpoint of OS was not met. The combination of atezolizumab and chemotherapy did not significantly outperform chemotherapy alone (16.0 vs. 13.4 months, *p* = 0.054).

Similarly, the KEYNOTE-361 trial [28] enrolled 1010 la/mUC patients to receive either pembrolizumab monotherapy, platinum-based chemotherapy, or a combination of both. After a median follow-up of 31.7 months, pembrolizumab added to chemotherapy did not substantially improve PFS or OS compared to chemotherapy alone. The mOS was similar across both the pembrolizumab and chemotherapy groups (15.6 vs. 14.3 months), even in patients with high PD-L1 expression (CPS ≥ 10%).

These trials highlight the challenges in enhancing the effectiveness of standard chemotherapy through the addition of immunotherapy in la/mUC. Despite initial hopes, combination therapy did not yield a significant improvement in OS, leading to a reevaluation of the roles of these immunotherapies in first-line treatment. Consequently, the current guidelines have become more restrictive, limiting the use of these immunotherapies to specific patient groups based on the PD-L1 expression and eligibility for platinum-based chemotherapy.

Fortunately, however, the phase 3, multinational, open-label CheckMate 901 trial finally marked a significant milestone in the treatment of unresectable or mUC [55]. This study demonstrated for the first time the efficacy of combining chemotherapy with immunotherapy in improving OS compared to chemotherapy alone.

The trial included 608 patients, with 304 in each group. At a median follow-up of 33.6 months, the results were significant. OS was notably longer in the group receiving the nivolumab combination, with a median survival of 21.7 months versus 18.9 months in the chemotherapy-only group. The hazard ratio for death was 0.78, indicating a 22% reduction in the risk of death with the nivolumab combination. PFS also favored the nivolumab group, with a hazard ratio of 0.72, reflecting a 28% reduction in the risk of disease progression or death. The mPFS was marginally longer in the nivolumab-combination group (7.9 months) compared to the chemotherapy-only group (7.6 months).

Moreover, the overall ORR was higher in the nivolumab-combination group at 57.6%, with a notable 21.7% of patients achieving a complete response, compared to 43.1% (complete response 11.8%) in the chemotherapy-only group. The median duration of complete response was also significantly longer with the nivolumab combination (37.1 months) than with chemotherapy alone (13.2 months). However, grade 3 or higher AEs were more frequent in the nivolumab-combination group (61.8%) compared to the chemotherapy-only group (51.7%).

It is unclear why the CheckMate 901 study [55] was the only one to yield positive results, unlike the other two studies [27, 28]. One reason might be that the only platinum agent used in CheckMate 901 was cisplatin, and only patients in better general condition without poorer kidney function (GFR < 60 ml/min) and poor ECOG PS (≧2) were selected. In patients treated with ICI in second-line therapy, cisplatin was associated with a better prognosis depending on the type of platinum agent used in the first line, but no difference was reported after adjusting for performance status and other parameters [56]. In other words, patients who could receive cisplatin were more likely to be in good general condition, which may have resulted in a higher therapeutic effect of immunotherapy as previously reported in 2nd line and more settings [22]. The NILE trial (NCT03682068) is a pivotal phase III study examining the efficacy of adding durvalumab, with or without tremelimumab, to standard chemotherapy in la/mUC. Its primary endpoint of OS is eagerly awaited as it could redefine the approach to upfront treatment in this disease.

### Combination of ADCs and ICIs for untreated mUC patients

A groundbreaking advancement in the treatment of la/mUC was achieved by combining antibody–drug conjugates (ADCs) with ICIs (Table 2) [9, 57]. ADCs, a novel class of therapeutics, consist of an antibody linked to a cytotoxic drug, allowing for targeted delivery of chemotherapy to tumor cells while minimizing systemic exposure and potential side effects. The trial in question focused on the efficacy of enfortumab vedotin (EV), an ADC targeting nectin-4, a protein highly expressed in UC cells [58]. EV is conjugated to a potent cytotoxic agent, which is already approved for la/mUC patients refractory to ICIs.

The phase IB/II EV-103/KEYNOTE-869 study provided the basis for accelerated FDA approval of this combination for la/mUC patients ineligible for cisplatin-containing chemotherapy [59]. In this multicohort study, the confirmed ORR was impressive at 73.3%, with 15.6% complete response in the dose escalation and dose expansion cohorts. Furthermore, the median duration of response was 25.6 months, underscoring the durability of this treatment approach.

The phase III EV-302/KEYNOTE-A39 trial compared EV combined with pembrolizumab against standard platinum-based chemotherapy (gemcitabine plus cisplatin or carboplatin) [60]. This trial evaluated the effectiveness and safety of this combination therapy, regardless of the patient’s cisplatin eligibility and PD-L1 expression status. The primary endpoints of the trial were PFS and OS. This combination markedly improved both PFS and OS compared to chemotherapy. The mPFS was extended to 12.5 months (compared to 6.3 months with chemotherapy), and the mOS reached 31.5 months (versus 16.1 months). The combination therapy also achieved a higher confirmed ORR of 67.7%. Although grade 3 treatment-related AEs were observed, they were generally manageable, with no new safety signals reported.

This trial not only marks a significant milestone in the treatment of la/mUC by successfully combining an ADC with an ICI but also establishes a new standard of care for first-line treatment in la/mUC, significantly improving patient outcomes with a manageable safety profile.

## Future directions

The emerging data from ongoing clinical trials in the realm of la/mUC are indicative of a promising future for combination therapies involving ADCs and ICIs [9]. These innovative treatments are poised to redefine the therapeutic landscape for la/mUC, where the unmet need for more effective and tolerable therapies remains significant (Table 3).

**Table 3:** Ongoing clinical trials of ADCs with ICIs for locally advanced or metastatic urothelial carcinoma patients

ICIs	ADCs	NCT number	Phase	Estimated primary completion date
First line				
Nivolumab/Ipilimumab	Sacituzumab govitecan	NCT04863885	I/II	2024/Apr
Toripalimab	Disitamab vedotin	NCT05302284	III	2026/Dec
Maintenance Therapy after induction chemotherapy				
Avelumab	Sacituzumab govitecan	NCT05327530	II	2026/Aug
Second line				
Atezolizumab	Enfortumab vedotin Sacituzumab govitecan	NCT03869190	IB/II	2024/Dec
Pembrolizumab	Disitamab vedotin	NCT04879329	II	2024/Oct

*ICIs* immune checkpoint inhibitor, *ADCs* antibody–drug conjugates, *NCT number* ClinicalTrials.gov identifier

The TROPHY-U-01 study of sacituzumab govitecan (SG) in la/mUC, with its different cohorts targeting various patient populations, has yielded encouraging results [61]. The outcome of Cohort 1 leading to accelerated FDA approval of SG indicates the efficacy of ADCs in patients who have progressed beyond standard platinum-based chemotherapy and ICIs. The promising results in Cohort 2, focusing on platinum-ineligible patients, further underscore the potential of ADCs in a broader patient population. Moreover, the combination of SG with pembrolizumab in Cohort 3, showing an ORR of 41% and mOS of 12.7 months, is particularly noteworthy. These results suggest that combining ADCs with ICIs can enhance therapeutic efficacy beyond what either approach can achieve independently. The ongoing phase III TROPiCS-4 trial (NCT04527991) comparing SG with chemotherapy after failure of initial treatments is expected to provide additional insights into the utility of ADCs in later treatment lines.

Parallel advancements in HER2-targeted therapies, another critical area, are also reshaping treatment strategies for la/mUC. HER2 gene amplification was identified in around 10% of la/mUC patients [62, 63]. The results from studies on disitamab vedotin [64] and upcoming trials, such as RC48G001 and phase III trials of disitamab vedotin combined with toripalimab versus chemotherapy, are setting the stage for more targeted approaches in HER2-expressing la/mUC.

Parallel to these trials, research is focusing on identifying biomarkers predictive of a response to ICIs. While PD-L1 expression is the most studied, emerging biomarkers such as tumor mutational burden, DNA damage repair alterations, tumor infiltration by CD8 T-cells, microsatellite instability-high status [65], the microbiome within digestive tracts [66, 67], localized tumor tissues [68], and blood [69] are gaining attention. The concept of a “urothelial carcinoma immunogram” is being developed to predict response to immunotherapy [70, 71] but is still in its nascent stage for clinical application.

Ongoing studies MORPHEUS-UC (NCT03869190) and JAVELIN Bladder Medley (NCT05327530) are investigating a variety of novel combinations, including ICIs with ADCs, PARP inhibitors, anti-CD47, anti-TIGIT, and cytokine receptor agonists. These trials aim to address resistance to single-agent immunotherapy and explore synergies between different classes of therapeutics.

## Conclusions

The landscape of la/mUC treatment is on the cusp of significant evolution, with numerous trials exploring combinations of ICIs with traditional and novel therapeutics. These studies aim to improve patient outcomes and also broaden the scope of effective treatments, marking an exciting era in the management of UC.
